# Diphenyl (benzyl­amido)phosphate

**DOI:** 10.1107/S1600536809053744

**Published:** 2009-12-19

**Authors:** Mehrdad Pourayoubi, Hossein Eshtiagh-Hosseini, Poorya Zargaran, Vladimir Divjakovic

**Affiliations:** a Department of Chemistry, Ferdowsi University of Mashhad, Mashhad 91779, Iran; b Department of Physics, Faculty of Sciences, University of Novi Sad, Trg D. Obradovica 3, 21000 Novi Sad, Serbia

## Abstract

The title compound, C_19_H_18_NO_3_P, was prepared by the reaction of diphenyl phospho­rochloridate and benzyl­amine. In the crystal structure, mol­ecules are linked *via* N—H⋯O=P hydrogen bonds into extended chains parallel to the *c* axis.

## Related literature

For related structures, see: Bao & Wulff (1993 ▶); Gholivand *et al.* (2005 ▶); Karolak-Wojciechowska *et al.* (1979 ▶).
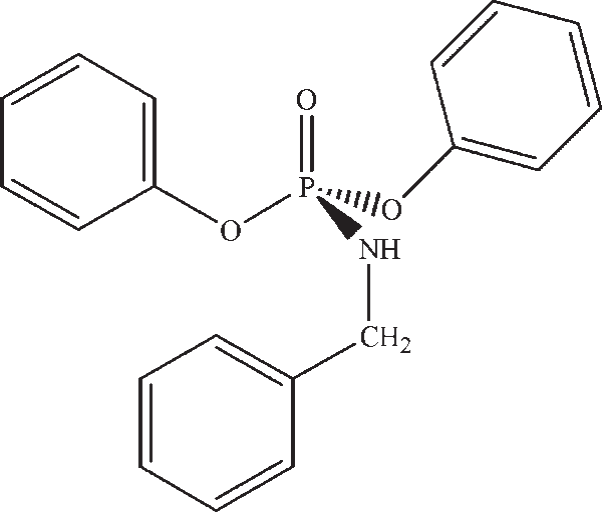


## Experimental

### 

#### Crystal data

C_19_H_18_NO_3_P
*M* *_r_* = 339.31
Monoclinic, 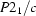

*a* = 10.0226 (5) Å
*b* = 19.2450 (8) Å
*c* = 10.2273 (5) Å
β = 115.375 (6)°
*V* = 1782.38 (17) Å^3^
*Z* = 4
Mo *K*α radiation
μ = 0.17 mm^−1^
*T* = 295 K
0.43 × 0.28 × 0.17 mm

#### Data collection

Oxford Diffraction Xcalibur diffractometer with a Sapphire3 (Gemini Mo) detector
Absorption correction: multi-scan *CrysAlis PRO* (Oxford Diffraction, 2009 ▶) *T*_min_ = 0.977, *T*_max_ = 1.000
8248 measured reflections
4105 independent reflections
2568 reflections with *I* > 2σ(*I*)
*R*_int_ = 0.019

#### Refinement

*R*[*F* ^2^ > 2σ(*F* ^2^)] = 0.040
*wR*(*F* ^2^) = 0.102
*S* = 0.91
4105 reflections
218 parameters
H-atom parameters constrained
Δρ_max_ = 0.19 e Å^−3^
Δρ_min_ = −0.31 e Å^−3^

### 

Data collection: *CrysAlis PRO* (Oxford Diffraction, 2009 ▶); cell refinement: *CrysAlis PRO*; data reduction: *CrysAlis PRO*; program(s) used to solve structure: *SIR92* (Altomare *et al.*, 1993 ▶); program(s) used to refine structure: *SHELXL97* (Sheldrick, 2008 ▶); molecular graphics: *Mercury* (Macrae *et al.*, 2008 ▶); software used to prepare material for publication: *SHELXL97*.

## Supplementary Material

Crystal structure: contains datablocks I, global. DOI: 10.1107/S1600536809053744/lh2963sup1.cif

Structure factors: contains datablocks I. DOI: 10.1107/S1600536809053744/lh2963Isup2.hkl

Additional supplementary materials:  crystallographic information; 3D view; checkCIF report

## Figures and Tables

**Table 1:** Hydrogen-bond geometry (Å, °)

*D*—H⋯*A*	*D*—H	H⋯*A*	*D*⋯*A*	*D*—H⋯*A*
N—H⋯O3^i^	0.86	1.97	2.8241 (15)	175

Symmetry code: (i) 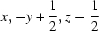.

**Fig. 1.**
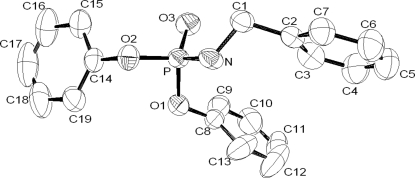
The molecular structure of the title compound, indicating the atom labeling scheme. Displacement ellipsoids are drawn at the 50% probability level.

**Fig. 2.**
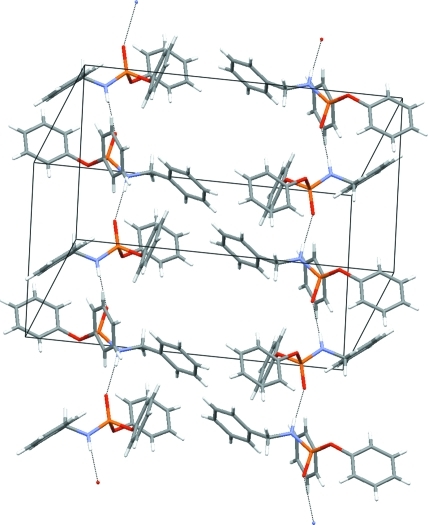
Part of the crystal structure with hydrogen bonds shown as dashed lines.

## supplementary crystallographic information

### Comment

In previous work, the synthesis and X-ray structures of some amidophosphoric acid ester compounds, such as [(C_6_H_5_)(CH_3_)CH—NH]P(O)(*p*—OC_6_H_4_CH_3_)_2_ (Gholivand *et al.*, 2005) and P(O)[OC_6_H_5_]_2_[N(CH_2_C_6_H_5_)(C(S)NHCH_2_C_6_H_5_)] (Karolak-Wojciechowska *et al.*, 1979) have been investigated. We report here on the synthesis and crystal structure of a new amido bis(phosphoric acid ester) compound, [C_6_H_5_—CH_2_—NH]P(O)[O—C_6_H_5_]_2_. The title compound was synthesized from the reaction of diphenyl phosphorochloridate with an excess amount of benzylamine. The P—O3 bond length of 1.4567 (10) Å and the P—N bond length of 1.5952 (14) Å are standard for this type of compound [for example for two crystallographically different [(C_6_H_5_)(CH_3_)CH—NH]P(O)(*p*—OC_6_H_4_CH_3_)_2_ molecules (Gholivand *et al.*, 2005), P═O = 1.462 (3) Å and 1.469 (3) Å and P—N = 1.610 (5) Å and 1.614 (5) Å and for the heterocyclic phosphorus compound obtained from sequential treatment of (+)2,2'-diphenyl-3,3'-biphenanthrol with phosphorus oxychloride and (S)-(-)-α-methylbenzylamine (Bao & Wulff, 1993) P═O = 1.456 (6) Å, P—N = 1.612 (7)]. In the title copmound, the P—O1 and P—O2 bond lengths are slightly different (1.5844 (12) Å and 1.5880 (12) Å) and the P atom has a distorted tetrahedral configuration (Fig. 1); the bond angles around the P atom are in the range of 98.80 (6)° [for the O1—P—O2 angle] to 114.84 (7)° [for the O3—P—O1 angle]. Molecules are linked *via* N—H···O═P hydrogen bonds (N···O3 = 2.8241 (15) Å) into extended chains parallel to the *c* axis (Fig. 2).

### Experimental

To a solution of diphenyl phosphorochloridate (0.572 g, 2.13 mmol) in chloroform (15 ml), a solution of benzylamine (0.456 g, 4.26 mmol) in chloroform (30 ml) was added at 273K. After 4 h of stirring, the solvent was evaporated in vacuum. The solid was washed with distilled water. Single crystals were obtained from a solution of the title compound in chloroform and n-heptane (4:1) after slow evaporation at room temperature. IR (KBr, cm^-1^): 3165 s, 2891 m, 2680 w, 2221 w, 1952 w, 1592 m, 1475 s, 1242 *vs*, 1198 *vs*, 1116 s, 1004 m, 931 *vs*, 759 s, 687 s.

### Refinement

H atoms were placed in the calculated positions and included in the refinement in a riding-model approximation with C-H = 0.93-0.97Å, N-H = 0.86Å and U_iso_(H) = 1.2U_eq_(C, N).

### Crystal data

**Table:**

C_19_H_18_NO_3_P	*F*(000) = 712
*M**_r_* = 339.31	*D*_x_ = 1.264 Mg m^−^^3^
Monoclinic, *P*2_1_/*c*	Mo *K*α radiation, λ = 0.71073 Å
Hall symbol: -P 2ybc	Cell parameters from 3418 reflections
*a* = 10.0226 (5) Å	θ = 3.2–29.1°
*b* = 19.2450 (8) Å	µ = 0.17 mm^−^^1^
*c* = 10.2273 (5) Å	*T* = 295 K
β = 115.375 (6)°	Prism, colorless
*V* = 1782.38 (17) Å^3^	0.43 × 0.28 × 0.17 mm
*Z* = 4	

### Data collection

**Table:**

Oxford Diffraction Xcalibur diffractometer with a Sapphire3 (Gemini Mo) detector	4105 independent reflections
Radiation source: Enhance (Mo) X-ray Source	2568 reflections with *I* > 2σ(*I*)
graphite	*R*_int_ = 0.019
Detector resolution: 16.3280 pixels mm^-1^	θ_max_ = 29.2°, θ_min_ = 3.2°
ω scans	*h* = −7→13
Absorption correction: multi-scan CrysAlis (Oxford Diffraction, 2009)	*k* = −17→24
*T*_min_ = 0.977, *T*_max_ = 1.000	*l* = −13→13
8248 measured reflections	

### Refinement

**Table:**

Refinement on *F*^2^	Secondary atom site location: difference Fourier map
Least-squares matrix: full	Hydrogen site location: inferred from neighbouring sites
*R*[*F*^2^ > 2σ(*F*^2^)] = 0.040	H-atom parameters constrained
*wR*(*F*^2^) = 0.102	*w* = 1/[σ^2^(*F*_o_^2^) + (0.0573*P*)^2^] where *P* = (*F*_o_^2^ + 2*F*_c_^2^)/3
*S* = 0.91	(Δ/σ)_max_ < 0.001
4105 reflections	Δρ_max_ = 0.19 e Å^−^^3^
218 parameters	Δρ_min_ = −0.31 e Å^−^^3^
0 restraints	Extinction correction: *SHELXL97* (Sheldrick, 2008), Fc^*^=kFc[1+0.001xFc^2^λ^3^/sin(2θ)]^-1/4^
Primary atom site location: structure-invariant direct methods	Extinction coefficient: 0.0047 (10)

### Special details

**Table:**

Experimental. #__ type_ start__ end____ width___ exp.time_ 1 omega -51.00 47.00 1.0000 19.0400 omega____ theta____ kappa____ phi______ frames - 21.0423 - 37.0000 300.0000 98#__ type_ start__ end____ width___ exp.time_ 2 omega 5.00 91.00 1.0000 19.0400 omega____ theta____ kappa____ phi______ frames - 21.0423 77.0000 150.0000 86#__ type_ start__ end____ width___ exp.time_ 3 omega -6.00 41.00 1.0000 19.0400 omega____ theta____ kappa____ phi______ frames - 21.0423 - 77.0000 240.0000 47
Geometry. All e.s.d.'s (except the e.s.d. in the dihedral angle between two l.s. planes) are estimated using the full covariance matrix. The cell e.s.d.'s are taken into account individually in the estimation of e.s.d.'s in distances, angles and torsion angles; correlations between e.s.d.'s in cell parameters are only used when they are defined by crystal symmetry. An approximate (isotropic) treatment of cell e.s.d.'s is used for estimating e.s.d.'s involving l.s. planes.
Refinement. Refinement of *F*^2^ against ALL reflections. The weighted *R*-factor *wR* and goodness of fit *S* are based on *F*^2^, conventional *R*-factors *R* are based on *F*, with *F* set to zero for negative *F*^2^. The threshold expression of *F*^2^ > σ(*F*^2^) is used only for calculating *R*-factors(gt) *etc*. and is not relevant to the choice of reflections for refinement. *R*-factors based on *F*^2^ are statistically about twice as large as those based on *F*, and *R*- factors based on ALL data will be even larger.

### Fractional atomic coordinates and isotropic or equivalent isotropic displacement parameters (Å^2^)

**Table:**

	*x*	*y*	*z*	*U*_iso_*/*U*_eq_	
P	0.97981 (4)	0.22655 (2)	0.06013 (4)	0.04171 (14)	
O1	0.87224 (12)	0.16886 (6)	−0.04116 (11)	0.0509 (3)	
O2	1.13289 (12)	0.18955 (6)	0.09457 (11)	0.0514 (3)	
O3	0.95622 (13)	0.24405 (6)	0.18721 (10)	0.0563 (3)	
N	0.97260 (16)	0.29192 (7)	−0.03860 (13)	0.0491 (4)	
H	0.9660	0.2836	−0.1238	0.059*	
C17	1.2659 (3)	0.00509 (17)	0.3197 (5)	0.1137 (12)	
H17	1.2997	−0.0363	0.3701	0.136*	
C2	0.84557 (18)	0.40475 (9)	−0.10133 (16)	0.0458 (4)	
C1	0.97616 (19)	0.36437 (9)	0.00303 (17)	0.0507 (4)	
H1A	1.0661	0.3856	0.0084	0.061*	
H1B	0.9782	0.3667	0.0986	0.061*	
C8	0.71826 (18)	0.17787 (9)	−0.09973 (17)	0.0490 (4)	
C14	1.17229 (17)	0.12655 (9)	0.17145 (18)	0.0495 (4)	
C13	0.6460 (2)	0.20013 (13)	−0.2392 (2)	0.0841 (7)	
H13	0.6973	0.2108	−0.2939	0.101*	
C3	0.7036 (2)	0.38616 (11)	−0.1264 (2)	0.0617 (5)	
H3	0.6885	0.3474	−0.0802	0.074*	
C7	0.8641 (2)	0.46219 (10)	−0.17277 (18)	0.0576 (5)	
H7	0.9585	0.4753	−0.1589	0.069*	
C9	0.6453 (2)	0.16092 (11)	−0.0180 (2)	0.0638 (5)	
H9	0.6972	0.1456	0.0768	0.077*	
C15	1.2344 (2)	0.12743 (12)	0.3197 (2)	0.0668 (5)	
H15	1.2451	0.1690	0.3697	0.080*	
C5	0.6047 (2)	0.48142 (13)	−0.2871 (2)	0.0779 (6)	
H5	0.5239	0.5074	−0.3487	0.094*	
C19	1.1552 (2)	0.06622 (12)	0.0974 (2)	0.0756 (6)	
H19	1.1122	0.0662	−0.0032	0.091*	
C6	0.7436 (2)	0.50035 (11)	−0.2647 (2)	0.0735 (6)	
H6	0.7576	0.5392	−0.3116	0.088*	
C10	0.4937 (2)	0.16685 (13)	−0.0781 (3)	0.0870 (7)	
H10	0.4423	0.1553	−0.0240	0.104*	
C18	1.2022 (3)	0.00495 (13)	0.1733 (5)	0.1065 (10)	
H18	1.1901	−0.0367	0.1234	0.128*	
C4	0.5843 (2)	0.42419 (14)	−0.2187 (2)	0.0765 (6)	
H4	0.4893	0.4109	−0.2347	0.092*	
C16	1.2806 (3)	0.06559 (18)	0.3931 (3)	0.0959 (9)	
H16	1.3221	0.0652	0.4937	0.115*	
C11	0.4196 (3)	0.18955 (14)	−0.2160 (4)	0.1061 (10)	
H11	0.3173	0.1937	−0.2562	0.127*	
C12	0.4943 (3)	0.20642 (15)	−0.2968 (3)	0.1170 (11)	
H12	0.4423	0.2222	−0.3912	0.140*	

### Atomic displacement parameters (Å^2^)

**Table:**

	*U*^11^	*U*^22^	*U*^33^	*U*^12^	*U*^13^	*U*^23^
P	0.0509 (2)	0.0414 (3)	0.0336 (2)	0.0017 (2)	0.01886 (17)	0.0016 (2)
O1	0.0523 (7)	0.0403 (7)	0.0545 (6)	0.0012 (5)	0.0175 (5)	−0.0030 (5)
O2	0.0500 (6)	0.0496 (8)	0.0554 (6)	0.0035 (6)	0.0234 (5)	0.0076 (6)
O3	0.0751 (8)	0.0621 (9)	0.0367 (6)	0.0050 (7)	0.0287 (5)	0.0045 (6)
N	0.0775 (9)	0.0403 (9)	0.0341 (6)	0.0033 (7)	0.0283 (6)	−0.0005 (6)
C17	0.0716 (17)	0.080 (2)	0.198 (4)	0.0297 (16)	0.067 (2)	0.068 (3)
C2	0.0540 (9)	0.0409 (10)	0.0455 (8)	−0.0013 (8)	0.0242 (7)	−0.0057 (8)
C1	0.0614 (10)	0.0417 (11)	0.0466 (9)	−0.0041 (9)	0.0210 (8)	−0.0025 (8)
C8	0.0525 (10)	0.0325 (10)	0.0494 (9)	−0.0007 (8)	0.0097 (8)	−0.0013 (8)
C14	0.0388 (8)	0.0452 (11)	0.0628 (11)	0.0030 (8)	0.0202 (8)	0.0040 (9)
C13	0.0884 (16)	0.0824 (17)	0.0522 (11)	−0.0214 (13)	0.0022 (10)	0.0109 (11)
C3	0.0655 (12)	0.0597 (13)	0.0704 (11)	−0.0050 (10)	0.0390 (10)	0.0001 (10)
C7	0.0605 (11)	0.0512 (12)	0.0630 (11)	−0.0032 (10)	0.0282 (9)	0.0037 (10)
C9	0.0561 (11)	0.0623 (14)	0.0643 (10)	−0.0019 (10)	0.0177 (9)	−0.0007 (10)
C15	0.0664 (11)	0.0729 (15)	0.0637 (11)	0.0193 (11)	0.0305 (9)	0.0141 (11)
C5	0.0683 (14)	0.0810 (18)	0.0751 (14)	0.0245 (13)	0.0216 (11)	0.0064 (13)
C19	0.0555 (11)	0.0584 (15)	0.0963 (15)	0.0033 (11)	0.0167 (10)	−0.0175 (13)
C6	0.0884 (16)	0.0574 (14)	0.0726 (13)	0.0109 (12)	0.0325 (12)	0.0164 (11)
C10	0.0579 (13)	0.0773 (18)	0.1166 (18)	−0.0056 (12)	0.0286 (13)	−0.0169 (15)
C18	0.0680 (15)	0.0452 (16)	0.190 (3)	0.0061 (13)	0.0401 (19)	−0.005 (2)
C4	0.0524 (11)	0.0902 (18)	0.0894 (14)	0.0068 (12)	0.0326 (11)	−0.0006 (14)
C16	0.0857 (16)	0.116 (2)	0.0997 (17)	0.0436 (17)	0.0529 (14)	0.0563 (19)
C11	0.0530 (13)	0.0567 (16)	0.153 (3)	−0.0022 (12)	−0.0092 (16)	0.0056 (17)
C12	0.093 (2)	0.090 (2)	0.0937 (18)	−0.0231 (16)	−0.0313 (15)	0.0330 (15)

### Geometric parameters (Å, °)

**Table:**

P—O3	1.4567 (10)	C3—C4	1.375 (3)
P—O1	1.5844 (12)	C3—H3	0.9300
P—O2	1.5880 (12)	C7—C6	1.381 (3)
P—N	1.5952 (14)	C7—H7	0.9300
O1—C8	1.4065 (19)	C9—C10	1.379 (3)
O2—C14	1.406 (2)	C9—H9	0.9300
N—C1	1.454 (2)	C15—C16	1.377 (3)
N—H	0.8600	C15—H15	0.9300
C17—C18	1.353 (4)	C5—C6	1.360 (3)
C17—C16	1.359 (4)	C5—C4	1.366 (3)
C17—H17	0.9300	C5—H5	0.9300
C2—C3	1.381 (2)	C19—C18	1.379 (4)
C2—C7	1.381 (2)	C19—H19	0.9300
C2—C1	1.503 (2)	C6—H6	0.9300
C1—H1A	0.9700	C10—C11	1.355 (4)
C1—H1B	0.9700	C10—H10	0.9300
C8—C13	1.363 (2)	C18—H18	0.9300
C8—C9	1.365 (3)	C4—H4	0.9300
C14—C19	1.356 (3)	C16—H16	0.9300
C14—C15	1.370 (2)	C11—C12	1.370 (4)
C13—C12	1.381 (3)	C11—H11	0.9300
C13—H13	0.9300	C12—H12	0.9300
			
O3—P—O1	114.84 (7)	C2—C7—H7	119.7
O3—P—O2	114.62 (6)	C6—C7—H7	119.7
O1—P—O2	98.80 (6)	C8—C9—C10	119.0 (2)
O3—P—N	113.69 (7)	C8—C9—H9	120.5
O1—P—N	107.77 (6)	C10—C9—H9	120.5
O2—P—N	105.75 (7)	C14—C15—C16	118.7 (2)
C8—O1—P	120.47 (10)	C14—C15—H15	120.7
C14—O2—P	121.58 (10)	C16—C15—H15	120.7
C1—N—P	125.61 (10)	C6—C5—C4	119.8 (2)
C1—N—H	117.2	C6—C5—H5	120.1
P—N—H	117.2	C4—C5—H5	120.1
C18—C17—C16	120.1 (3)	C14—C19—C18	119.2 (2)
C18—C17—H17	120.0	C14—C19—H19	120.4
C16—C17—H17	120.0	C18—C19—H19	120.4
C3—C2—C7	118.08 (17)	C5—C6—C7	120.3 (2)
C3—C2—C1	120.83 (16)	C5—C6—H6	119.8
C7—C2—C1	121.08 (16)	C7—C6—H6	119.8
N—C1—C2	112.54 (13)	C11—C10—C9	119.9 (2)
N—C1—H1A	109.1	C11—C10—H10	120.1
C2—C1—H1A	109.1	C9—C10—H10	120.1
N—C1—H1B	109.1	C17—C18—C19	120.4 (3)
C2—C1—H1B	109.1	C17—C18—H18	119.8
H1A—C1—H1B	107.8	C19—C18—H18	119.8
C13—C8—C9	122.16 (18)	C5—C4—C3	120.2 (2)
C13—C8—O1	118.58 (17)	C5—C4—H4	119.9
C9—C8—O1	119.17 (15)	C3—C4—H4	119.9
C19—C14—C15	121.14 (19)	C17—C16—C15	120.5 (3)
C19—C14—O2	119.20 (16)	C17—C16—H16	119.7
C15—C14—O2	119.57 (17)	C15—C16—H16	119.7
C8—C13—C12	117.9 (2)	C10—C11—C12	120.5 (2)
C8—C13—H13	121.0	C10—C11—H11	119.8
C12—C13—H13	121.0	C12—C11—H11	119.8
C4—C3—C2	120.91 (19)	C11—C12—C13	120.5 (2)
C4—C3—H3	119.5	C11—C12—H12	119.7
C2—C3—H3	119.5	C13—C12—H12	119.7
C2—C7—C6	120.63 (18)		
			
O3—P—O1—C8	56.68 (13)	C3—C2—C7—C6	1.0 (3)
O2—P—O1—C8	179.11 (11)	C1—C2—C7—C6	−177.78 (16)
N—P—O1—C8	−71.14 (13)	C13—C8—C9—C10	−0.4 (3)
O3—P—O2—C14	58.59 (14)	O1—C8—C9—C10	−177.10 (18)
O1—P—O2—C14	−63.99 (12)	C19—C14—C15—C16	0.5 (3)
N—P—O2—C14	−175.37 (11)	O2—C14—C15—C16	−176.02 (16)
O3—P—N—C1	13.52 (17)	C15—C14—C19—C18	−0.5 (3)
O1—P—N—C1	142.00 (14)	O2—C14—C19—C18	176.05 (17)
O2—P—N—C1	−113.09 (14)	C4—C5—C6—C7	−0.3 (3)
P—N—C1—C2	−124.33 (14)	C2—C7—C6—C5	−0.6 (3)
C3—C2—C1—N	60.3 (2)	C8—C9—C10—C11	−0.3 (4)
C7—C2—C1—N	−120.96 (17)	C16—C17—C18—C19	1.7 (4)
P—O1—C8—C13	99.80 (18)	C14—C19—C18—C17	−0.6 (3)
P—O1—C8—C9	−83.34 (19)	C6—C5—C4—C3	0.7 (3)
P—O2—C14—C19	99.71 (17)	C2—C3—C4—C5	−0.2 (3)
P—O2—C14—C15	−83.73 (17)	C18—C17—C16—C15	−1.7 (4)
C9—C8—C13—C12	1.0 (3)	C14—C15—C16—C17	0.6 (3)
O1—C8—C13—C12	177.8 (2)	C9—C10—C11—C12	0.3 (4)
C7—C2—C3—C4	−0.7 (3)	C10—C11—C12—C13	0.4 (4)
C1—C2—C3—C4	178.17 (17)	C8—C13—C12—C11	−1.1 (4)

### Hydrogen-bond geometry (Å, °)

**Table:**

*D*—H···*A*	*D*—H	H···*A*	*D*···*A*	*D*—H···*A*
N—H···O3^i^	0.86	1.97	2.8241 (15)	175

Symmetry codes: (i) *x*, −*y*+1/2, *z*−1/2.
